# Part 2: The feasibility of utilising photovoice method and the World Health Organization Quality of Life instrument in evaluating the Community-Based Rehabilitation programme in Namibia: A pilot study

**DOI:** 10.4102/ajod.v7i0.419

**Published:** 2018-11-01

**Authors:** Tonderai W. Shumba, Indres Moodley

**Affiliations:** 1 Discipline of Public Health Medicine, University of KwaZulu-Natal, South Africa

## Abstract

**Background:**

Evaluation of Community-Based Rehabilitation (CBR) programmes in Namibia has been primarily quantitative, focusing mainly on outputs, including numbers of persons with disabilities served, referrals made and activities implemented. Little or no evidence is available on experiences and quality of life of persons with disabilities, despite the CBR programme being operational for more than 20 years. The 2011 World Report on Disability recommended the use of appropriate tools to fill the research gap by integrating the experiences of persons with disabilities and their quality of life.

**Objectives:**

The overall objective of the larger cohort study is to develop a monitoring and evaluation tool that can measure and integrate the experiences of persons with disabilities and their quality of life within the context of the CBR Programme in Namibia.

**Method:**

An adapted photovoice process was conducted with six purposively selected participants over a period of 1 month. The World Health Organization Community-Based Rehabilitation (WHO CBR) Matrix was used to identify the themes and subthemes. Participants were requested to complete the World Health Organization Quality of Life (abbreviated version) (WHOQOL-BREF) instrument at the end of the photovoice process to determine their quality of life.

**Results:**

Administering the WHOQOL-BREF instrument at the end of the photovoice process measured both the quality of life of persons with disabilities and at the same time indicated the convergence and divergence in the two data collection methods. The study demonstrated a stronger convergence than divergence of the two methods. A feasibility criterion was mapped for future studies.

**Conclusion:**

This study demonstrated that photovoice is a flexible method that can be used with a variety of disabilities and has the potential of being combined with the WHOQOL-BREF assessment form. A larger cohort study may consider implementing photovoice and WHOQOL-BREF on multiple study sites and be able to compare results, considering geographical and demographic variables. The feasibility of utilising each method alone and in combination offered valuable insights on future conceptual framing of CBR programme evaluation. This conceptual framing will allow CBR practitioners to appreciate how these two methods contribute to a rigorous process of CBR programme evaluation.

## Background

Community-Based Rehabilitation (CBR) is a strategy designed to improve service delivery, social integration and quality of life and to protect and promote the human rights of persons with disabilities (WHO 2011). Community-Based Rehabilitation was introduced to Africa in the early 1980s and was initiated in Namibia in 1992 (Ministry of Health and Social Services 2013) and formally adopted in 1997 (Government Republic of Namibia 1997). Since then, CBR implementation and evaluations have been affected by the evolving global trends in models of disability. CBR has evolved from healthcare service delivery to community development (WHO, UNESCO & ILO 2004). The adoption of the United Nations Convention on the Rights of Persons with Disabilities (UNCRPD) in 2006 affirmed the need to uphold human rights by mandating member states to promote and protect the rights of persons with disabilities (UN 2006). Furthermore, the CBR Guidelines (WHO et al. 2010) added a new rights-based approach with an emphasis on inclusion and active participation of persons with disabilities. As a result of the evolving concept of CBR, it has been implemented in various contexts thus affecting the global scope on CBR evaluations (M’kumbuzi & Myezwa 2016).

While the CBR Programme has been now rolled out to all 14 regions in Namibia, there are still districts where it is yet to be implemented. Evaluation of CBR Programmes has been quantitative, focusing mainly on outputs such as numbers of persons with disabilities served, referrals made and activities that are carried out (Ministry of Health and Social Services 2013). Little or no evidence is available on experiences of persons with disabilities, despite the CBR Programme being operational for more than 20 years. Insights into experiences and understandings of those who are ‘exposed’ to CBR can best be obtained through qualitative methods. Recently, Shumba and Moodley (2017a) in their assessment of the implementation of the CBR disability programmes identified the need for a qualitative evaluation tool that can effectively elicit the experiences of persons with disabilities on the CBR Programme.

The World Health Organization Community-Based Rehabilitation (WHO CBR) Guidelines (WHO et al. 2010) state that the main objective of CBR is to improve the quality of life of persons with disabilities. The measurement of quality of life becomes critical in providing an additional measure to corroborate the elicited experiences of persons with disabilities. This observation is supported by the 2011 World Report on Disability that recommended the use of appropriate tools to fill the research gap by integrating the experiences of persons with disabilities and their quality of life.

Under the aegis of Article 32 of the UNCRPD, persons with disabilities should be consulted in services in which they are involved (UN 2006). Similarly, Madden et al. (2015) advocated for monitoring systems that are participatory and community owned to ensure programme quality and sustainability. Photovoice is one method that challenges the established paradigms of representation by enabling vulnerable groups to effectively communicate their experiences and needs. Photovoice was developed by Wang and Burris in 1997 to enable vulnerable people to express and reflect their experiences with the aid of photographs and to formulate concerns that can be communicated to implementers and policymakers.

Although photovoice has been used widely with persons with disabilities and other studies (HIV, tuberculosis, etc.) in Africa, it has limitedly been used as a research method with persons with disabilities in southern Africa (Shumba & Moodley 2017b). This is possibly attributed to lack of knowledge on the application of the method or limited value placed on monitoring and evaluation of disability programmes. Thus, there is a gap in knowledge on the application of photovoice as a disability evaluation tool particularly with persons with disabilities participating in CBR Programmes. This was reported in the scoping study conducted by Shumba and Moodley (2017b) that provided some insights on photovoice application for evaluation of CBR Programmes.

Because photovoice does not directly measure quality of life, there is need for a complementary tool that can measure quality of life. One such tool is the World Health Organization Quality of Life instrument (WHOQOL-BREF) which is an abbreviated version of the WHOQOL-100 and was developed to assess the ‘individual’s perceptions of their position in life in the context of the culture and value systems in which they live and in relation to their goals, expectations, standards and concerns*’* (World Health Organization 1998:1). The WHOQOL-BREF instrument has been proposed to have uses in establishing baseline scores, identifying changes in quality of life, research and policy making (World Health Organization 1998). Furthermore, the WHOQOL-BREF instrument measures other aspects including sexual health that in most instances is not measured by other quality of life instruments. Evaluating the effects of CBR Programme on quality of life of persons with disabilities can further help to prioritise areas for more effective use of resources, especially in resource-limited settings including Namibia. Studies on the evaluation of CBR Programmes (Grandisson, Hébert & Thibeault 2014) advocate for mixed methods and participatory tools to empower persons with disabilities to effectively communicate their needs to programme implementers and policymakers.

Based on the above background, it was therefore important to assess the feasibility of utilising photovoice in conjunction with WHOQOL-BREF instrument before wider application.

## Methodology

### Objectives

#### Objective of the main study

The overall objective of the larger cohort study is to develop a monitoring and evaluation tool that can measure and integrate the experiences of persons with disabilities and their quality of life within the context of the CBR Programme in Namibia. This pilot study will be used to identify any challenges that may need to be addressed in a larger cohort study.

#### Specific objectives of the pilot study

- To assess the feasibility of the processes that are key to implementing photovoice in conjunction with the WHOQOL-BREF instrument.
- To identify potential human and data management problems.
- To assess the ethical issues, trustworthiness and responsiveness of photovoice and the WHOQOL-BREF assessment.

### Feasibility criteria

The assessment of the outcomes of the feasibility success was based on the objectives. Four broad classifications (process, resources, management and scientific) (Van Teijlingen et al. 2001) were adapted and utilised to map the outcomes of the pilot study (Table 1).

**TABLE 1:** Feasibility criteria.

Main reason	Issue assessed
1. Process: Assessing the feasibility of the processes that are key to photovoice and WHOQOL-BREF instrument	Determine selection of: – participants: persons with physical disabilities including caregivers or family of persons with physical disabilities and visual impairments – research assistants: CBR experience and language competence Eligibility criteria for participants – is it adequate or too restrictive or broad Critical issues to address in retention of participants Understanding the contents and administration of data collection tools – photovoice technique and WHOQOL-BREF instrument Adherence to photovoice ethical issues
2. Resources: Forecasting time and resource problems that can occur during the larger cohort study	Determining process time from photography assignment to photo gallery Ascertaining time needed to fill out the WHOQOL-BREF instrument Establishing the type and quantity of language versions required of the WHOQOL-BREF instrument Determine the need for sign language interpreters in the case of persons with hearing impairment Establishing quantity and cost of Braille or large print instruments if needed Determining the best type of camera to use, either disposable or digital camera based on cost, availability and processing Establishing contingency plans in case participants’ cameras are broken or lost before processing Establishing the nearest possible place for processing digital cameras and the cost Estimating the cost and time needed for the researcher to travel to the research sites Identifying the transport needed
3. Management: Establishing potential human and data management problems	Identifying the challenges of research assistants in managing participants during both the photovoice process and assisting with filling out or completion of WHOQOL-BREF Identifying challenges of participants during photovoice process and in completing the WHOQOL-BREF Establishing problems in processing cameras Determining safe places to keep cameras before processing Establishing how to deal with issues of confidentiality of photographs before photo gallery and publication Estimating the number of photography assignments needed before data are refined and ready for photo gallery
4. Scientific: Assessment of ethical issues, trustworthiness, response to photovoice and WHOQOL-BREF instrument	Determine the ethical and trustworthiness issues pertaining to photovoice and persons with disabilities Establish whether photovoice and WHOQOL-BREF instrument can elicit experiences and quality of life of persons with disabilities, respectively Establish sample size needed Determine whether or not to include caregivers or family of persons with physical disabilities Determine other types of disabilities that can be investigated using photovoice and WHOQOL-BREF instrument Estimate duration of study Propose the best way of combining photovoice and WHOQOL-BREF instrument in evaluating CBR Programme

*Source*: Adapted from Adapted from Van Teijlingen, E.R, Rennie, A.M, Hundley, V. & Graham, W., 2001, ‘The importance of conducting and reporting pilot studies: The example of the Scottish births survey’, *Journal of Advanced Nursing* 34, 289–295. https://doi.org/10.1046/j.1365-2648.2001.01757.x

WHOQOL-BREF, World Health Organization Quality of Life instrument; CBR, Community-Based Rehabilitation.

### Study site

The pilot study was conducted in the rural settlement of Groot Aub in the Khomas Region which is located 45 kilometres from the capital city Windhoek and is easily accessible for data collection. This site was chosen as it has a similar composition to the ones to be chosen for the larger cohort study with respect to variables such as the types of disability, geographic distribution of participants, geographic terrain and cultural diversity. The Groot Aub CBR Programme was initiated in 2015 and the main activities include identification and screening of persons with disabilities, home visits and referral for services. The CBR Programme consists of 15 CBR volunteers who were trained utilising the WHO CBR Matrix (WHO et al. 2010).

### Participants

The population of this pilot study included all persons with disabilities and their caregivers who are participating in, or are beneficiaries of, the CBR Programme in Groot Aub. The inclusion of caregivers as participants was based on the scoping review (Shumba & Moodley 2017b) that revealed that caregivers can also be utilised to investigate life experiences of persons with disabilities particularly those with multiple and severe disabilities. Purposive sampling utilising a research assistant was used to identify eight participants. These participants had the following characteristics: 18 years or older, involved with the CBR Programme for at least 1 year and able to read and speak basic English. Persons with intellectual disabilities or mental illness or emotional disorders were excluded. The research assistant chosen was the Senior Community Liason Officer who is the Regional Community Liason Officer of the Khomas Region under the Office of the Presidency, Department of Disability Affairs.

Initially 15 participants were invited to participate in the photovoice process, but only eight met the inclusion criteria. Of these eight participants, six completed the study. One of the six participants opted out from group interviewing and requested self-representation of concerns with the local councillor. The participant needed privacy and confidentiality of self-expression for fear of retribution.

### Intervention methods

Intervention methods included two phases, namely the implementation of the photovoice method and the administration of the WHOQOL-BREF assessment.

#### Phase 1: Photovoice method

**Training:** A photovoice training was conducted with the eight participants prior to data collection. This half-day training included the following aspects: photovoice process, group objectives, informed consent to participate, how to use the camera, the basics on how to take photographs, potential subject matter and themes of photographs and ethical considerations when photographing human subjects. This training also served as a platform for participants to introduce themselves and get to know each other.

The researcher utilised a structured technique as suggested by Wang (1999) to guide the participants to identify aspects relevant to their CBR experience. The structured photovoice technique is known by an acronym called ‘SHOWeD’ as explained below:

SHOWeD

*What do we* ***S****ee here? What is really* ***H****appening here? How does this relate to* ***O****ur lives?* ***W****hy does this situation, concern or strength* ***E****xist? What can we* ***D****o about it?* (Wang 1999)

This technique enabled and empowered the participants to capture relevant photographs and critically analyse content regarding their experiences. Furthermore, the researcher explained that the technique would assist in codifying and selecting themes and subthemes during the follow-up individual interviews.

For data collection, each participant was then issued with the following materials: one disposable camera, consent forms for the photographed subjects to sign verifying consent to be photographed as well as to publish the photographs.

**Data collection:** This part of the project involved taking of photographs by the participants, collection of the cameras and signed consent forms, development of photographs and reflection and was completed in 1 week. Participants were allowed 6 days to take their photographs and to return their cameras and the subject release forms to the research assistant who then sent them to the researcher. About midway through the time period, the research assistant telephonically reminded the participants of their deadlines and offered encouragement and advice where necessary. After 6 days, the researcher received the cameras and then had the photographs processed which took 1 day. Each participant’s set of photographs are saved and coded on separate compact discs (CDs). This master set of CDs was retained in safe-keeping by the researcher.

**Data analyses:** During week two, the researcher and research assistant returned with the processed photographs to Groot Aub. Participants were requested to come to the Councillor’s office at selected times over a period of 3 days for individual interviews, selection of ‘best’ photographs, codification and feedback on the photography assignment. The researcher requested each participant to contextualise each photograph using the ‘SHOWeD’ technique (Wang 1999). Each participant was allocated time for individual selection of the ‘best’ 10 photographs to describe their experiences regarding the Groot Aub CBR Programme. The researcher requested each participant to categorise photographs with similar meaning together and identify a theme. Those belonging to one theme, but of a subcategory were assigned to a subtheme. Participants were then requested to give feedback on the challenges and achievements they had during the photovoice process. A trusting or safe atmosphere allowed participants to express themselves freely. Participants also shared thoughts on other photographs they wished they could have taken, but did not.

On completion of individual feedback sessions, participants were invited to a group discussion to share their selected photographs with the other participants. The aim of the group discussion was to select the final photographs that best represented a collective story for the CBR Programme. In light of the principle of self-determination and self-representation, participants were informed that the group discussion was voluntary and they had the option not to share their stories in a larger group. Earlier it is stated that one participant declined.

Before the group discussion, participants were encouraged to circulate through the room to view and reflect on all the photographs that were displayed and to talk with other participants about their experience of taking and selecting photographs. Simultaneously, the researcher circulated through the room to communicate with participants and ask probing questions, taking field notes. The researcher then used an LCD projector to display the selected photographs of each participant. Each participant was allocated 20 min to present their findings and instructed to link specific pictures with each of their identified themes. The other participants were requested to refrain from asking questions during this process; they could however make a note of their questions and their own related stories to share in the final discussions.

Group coding was done through full group discussion to develop collective themes. At this stage, all participants could share their individual and collective experiences as they related to specific photographs, revising the underlying issues and themes. The researcher asked probing questions to guide the analysis of the data. The researcher facilitated the process of grouping the themes and subthemes utilising the WHO CBR Matrix (WHO et al. 2010) as a conceptual framework. Participants took ownership and actively engaged in the discussions and the thematic analysis. Final themes and subthemes were then identified and photographs to present these themes were collectively agreed upon. The researcher deemed the data saturated when no new statements, regarding the meaning of the photographs, were made and all the participants reached an agreement on what was discussed. The researcher recorded the discussion and took field notes of participants’ responses. The discussions and the workshop were closed with some discussion questions: 1) What is the best way to present the findings? Do you prefer a photo gallery or a poster release or presentation?, 2) Can photovoice be implemented on a larger scale in other CBR Programmes around the country? and 3) Suggest improvements to the photovoice process.

On completion of the group discussion, participants were invited to share their experiences and photographs either by preparing a photo gallery or a poster presentation. Participants were informed that this was voluntary, and they had the right to refuse and represent themselves individually. However, the power of combined effort with one voice was emphasised. As a result of limited funds and time to organise a photo gallery, the consensus of the group was to produce a poster with the exception of one participant who requested an individual meeting or consultation with the local Councillor. Each participant was then provided with a CD with all photographs and hard copies of the photographs to distribute to the subjects they used as a token of appreciation. The participants were encouraged to develop a work plan in cooperation with the CBR Committee to tackle some of the issues of concern with assistance from the research assistant.

#### Phase 2: World Health Organization Quality of Life-BREF assessment

**Orientation of the World Health Organization Quality of Life instrument:** Upon completion of the photovoice process, the researcher invited all participants to complete the WHOQOL-BREF instrument (Appendix 6). The researcher gave a brief overview of the WHOQOL-BREF instrument. Furthermore, the researcher explained that the purpose of completion of the WHOQOL-BREF instrument was to provide preliminary feedback on participants’ and research assistant’s understanding of WHOQOL-BREF instrument, human and data management problems, resource implications and the feasibility of combining the WHOQOL-BREF instrument and the photovoice method.

**Data collection:** The participants were requested to complete the WHOQOL-BREF assessment based on their life experiences during the past two years. Because the main languages for Khomas Region are English and Afrikaans, the researcher administered the internationally translated English and Afrikaans versions of the WHOQOL-BREF instrument. The researcher supervised the administration of the WHOQOL-BREF and offered clarity to participants when necessary.

**Data analyses:** Data for this study were manually calculated following the steps and formulas stipulated by WHOQOL-BREF Instructions Manual (WHOQOL-BREF Group 1996). Manual calculations for each participant are shown on page 1 of the WHOQOL-BREF Instructions Manual (Appendix 1). Converting the domain scores to transformed scores (comparable with WHOQOL-BREF-100 [4–20 scale] and [0–100 scale]) is shown in Table 4 on page 11 of the WHOQOL-BREF Instructions Manual (WHOQOL-BREF Group 1996).

### Trustworthiness

Trustworthiness was ensured using Lincoln and Guba’s strategies for the qualitative approach including credibility, transferability, dependability and confirmability (Lincoln & Guba 1985). Credibility was assured by prolonged engagement in photography assignment until the scope of data is adequately covered and data saturation of experiences of participants on the CBR Programme was obtained; referential adequacy through the use of cameras and field notes, triangulation through engagement in individual interviewing, group interviewing and photography, member checks on participants’ responses and peer debriefing of themes with research assistant and second author were also applied. Purposive sampling and dense descriptions ensured transferability. Photographs, written field notes, methodological notes and reflexivity ensured dependability and confirmability.

### Ethical considerations

Ethics approval was obtained from the Human Sciences Ethics Research Committee of the University of KwaZulu-Natal (Reference No: HSS/0646/015D) and the Ministry of Health and Social Services in Namibia (17/3/3). A full disclosure of the purpose of the pilot study was completed including the definition of a pilot study, the objectives of the larger cohort study, the objectives of the pilot study and the criteria for success of feasibility (Appendix 1). Furthermore, the researcher trained the participants to request consent from subjects to be photographed and to publish the photographs for use in conferences, seminars, journals or books (Appendix 2, 3 and 4). Noting the ethical considerations of the photovoice method, the participants were trained on the key concerns and their expected practice during the photovoice process (Appendix 5).

## Results

### Participants characteristics

Of the six participants that were included five were persons with disabilities and one was a caregiver to persons with disability. The characteristics of participants are shown in Table 2.

**TABLE 2:** Participant characteristics.

Participant code	Type of disability or caregiver	Age	Gender	Employment status	Highest education received
P1	Physical (paraplegia)	39	Male	Not employed	None
P2	Visual impairment	28	Female	Not employed	Secondary school
P3	Physical (stroke)	46	Female	Not employed	Secondary school
P4	Caregiver	46	Female	Not employed	Secondary school
P5	Physical (impaired limb)	29	Male	Not employed	Secondary school
P6	Physical (poliomyelitis)	21	Male	Not employed	None

### Baseline results

#### Photovoice baseline results

A number of key themes and subthemes were elicited from experiences of the participants using the photovoice methods. The final themes and subthemes collectively represent the experiences of the participants regarding the CBR Programme. It is important to note that although the themes were relevant across the participants, individual differences may still be important depending on the emphasis each individual placed on each theme. The themes and subthemes with accompanying photographs are discussed below.

**Key theme 1: Secure livelihood**

**FIGURE 1:**
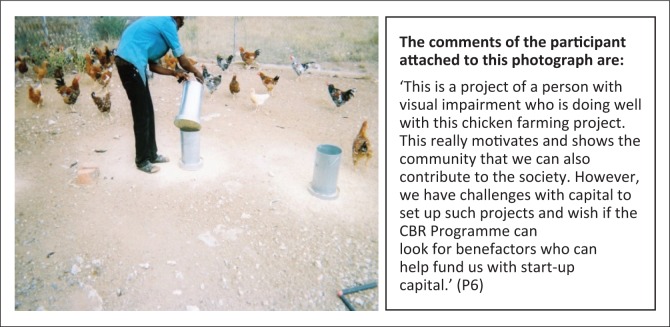
Need for start-up capital for projects. Person with visual impairment who owns a chicken farming project.

***Subtheme 1: Self-employment:*** Notwithstanding the stigma that persons with disabilities are not capable or productive, it was reported that some per sons with disabilities are setting up viable income-generating projects (Figure 1).

**FIGURE 2:**
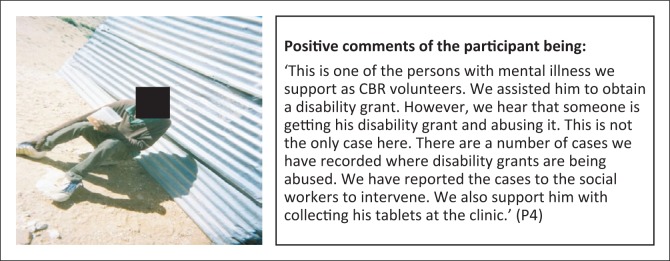
Advocacy for disability grants. Person with mental illness supported by Community-Based Rehabilitation Programme to obtain disability grant.

**FIGURE 3:**
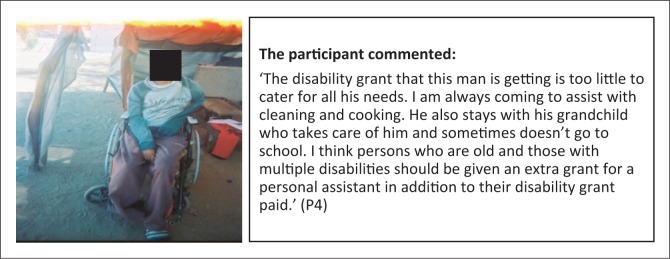
Personal assistance required for persons with multiple and severe disabilities. Person with disability benefitting from the Community-Based Rehabilitation Programme through provision of cleaning and cooking support.

**Subtheme 2: Social protection:** The CBR Programme has been assisting persons with disabilities to access disability grants, but some recipients of the grants are not utilisi ng the funds well or are experiencing exploitation by others (Figure 2). However, the disability grant is not adequate for some persons with multiple disabilities or the elderly with disabilities, as in some cases they need funds to pay their personal assi stants and other supplies and materials specific to their disability (Figure 3).

**Key theme 2: Health services**

**FIGURE 4:**
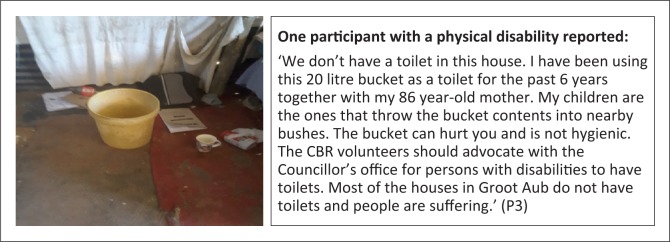
No toilet at home. Bucket serving as a toilet for an 86-year-old woman.

**FIGURE 5:**
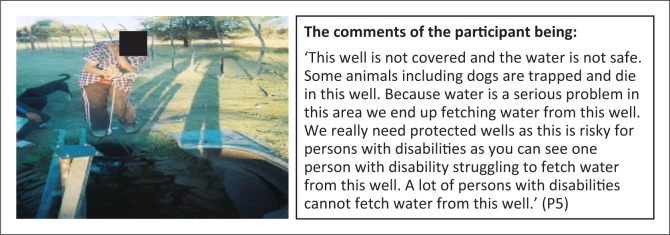
Risky and unhygienic water sources. Person with disability fetching water from an unprotected well.

**Subtheme 1: Water and sanitation:** A majority of the participants (4) reported challenges of not having adequate toilets, to the extent that they u se buckets as toilets (Figure 4). Furthermore, access to clean water is also a challenge in Groot Aub, exposing persons with disabilities to risks from using water from unsafe sources (Figure 5).

**FIGURE 6:**
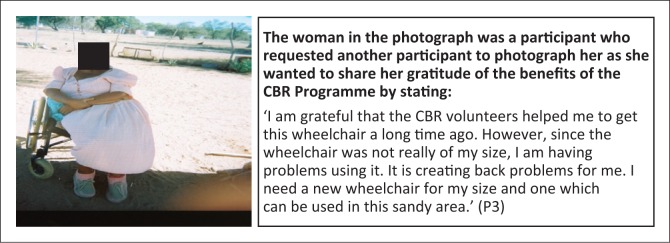
Inappropriate wheelchair size can cause back pain. CBR Programme advocating for a woman with stroke in need of a wheelchair of the right size.

**Subtheme 2: Assistive devices:** There is a critical shortage of wheelchairs creating a situation in which persons with disabilities are receiving donated wheelchairs which do not meet their specialised needs and measurements (Figure 6). Although CBR is being recognised as a vehicle to assist persons with disabilitie s to access wheelchairs, there is still a lack of expertise in measuring and providing wheelchairs with the required specifications. Recipients of wheelchairs need to be measured by a qualified Occupational Therapist or medical rehabilitation worker to pre vent further musculoskeletal and neurological complications.

**FIGURE 7:**
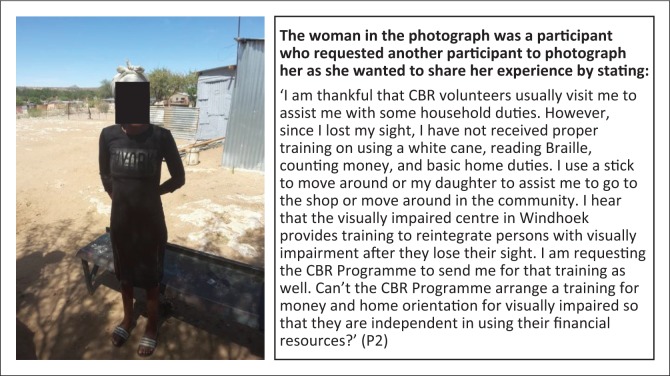
Rehabilitation in mobility, Braille, money counting and household duties critical for persons with visual impairments. Woman with visual impairment in need of rehabilitation services.

**Subtheme 3: Rehabilitation:** Five participants reported challenges with access to rehabilitation services. Figure 7 depicts comments of one of these participants.

**Key theme 3: Accessibility**

**FIGURE 8:**
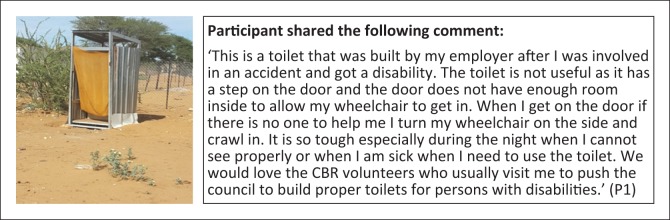
Need for physical accessibility to toilets. Toilet was built for person with a disability, but accessibility to a wheelchair was not considered.

**FIGURE 9:**
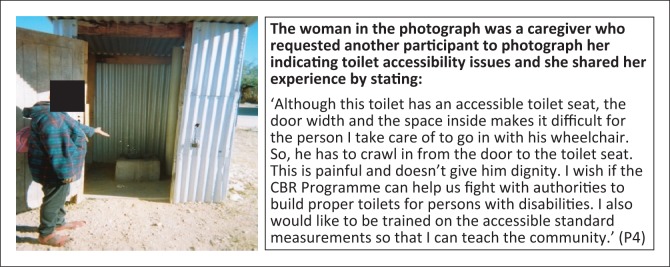
Community knowledge gap on required door width of toilets. Toilet with accessible toilet seat but inaccessible to wheelchair.

**Subtheme 1: Physical accessibility:** Physical accessibility was noted as one of the major challenges facing persons with disabilities in Groot Aub with some household reporting that toilets were not accessible to wheelchairs (Figure 8). However, some toilet s constructed have elements of accessibility as a result of the efforts of CBR volunteers, but are still not fully accessible, as indicated by the height of the seat in the photograph (Figure 9).

**FIGURE 10:**
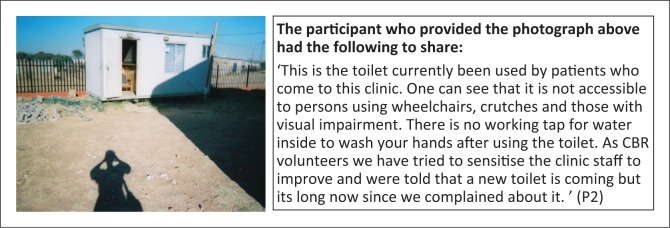
Inaccessible public buildings. Need for accessible toilet at local clinic.

**FIGURE 11:**
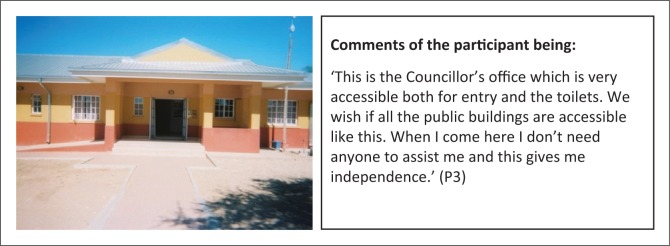
Leading by example. Constituency Councillor’s office with proper physical accessibility.

Physical accessibility to buildings is also a problem at public services including schools, clinic and police station (Figure 10). Correspondingly, a positive experience regarding physical accessibility to public buildings was recorded (Figure 11).

**FIGURE 12:**
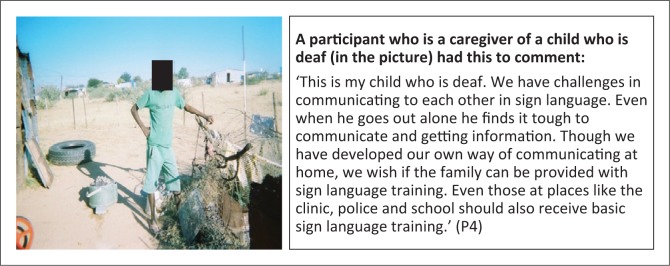
Advocacy for sign language training for family and community. Deaf child with communication challenges with family and community.

**Subtheme 2: Information accessibility:** Most persons with disabiliti es, particularly those with visual impairment (Figure 7) and the deaf (Figure 12) face challenges in accessing information. User-sensitive and friendly modes of communication need to be considered.

#### WHOQOL-BREF instrument baseline results

**World Health Organization Quality of Life domain raw scores and transformed scores:** The WHOQOL-BREF instrument was administered to the six participants. The results of participants’ responses regarding quality of life scores determined by the four WHOQOL-BREF instrument domains are shown in Table 3 and integration of results of photovoice method and WHOQOL-BREF instrument per participant are shown in Table 4.

**TABLE 3:** Participants’ World Health Organization Quality of Life transformed scores on scale 0–100.

Participant code	Domain 1: Physical health	Domain 2: Psychological	Domain 3: Social relationships	Domain 4: Environment
P1	44	50	44	31
P2	69	56	19	31
P3	44	56	69	44
P4	56	38	44	31
P5	63	88	50	56
P6	50	69	31	50

*Source*: Adapted from WHOQOL-BREF Group, 1996, *WHO-BREF instrument -Introduction, administration, scoring and version of the assessment*, World Health Organisation, Geneva.

**TABLE 4:** Combined participants` and World Health Organization Quality of Life transformed scores and photovoice responses.

Participant code	Domain 1: Physical health (0–100)	Domain 2: Psychological (0–100)	Domain 3: Social relationships (0–100)	Domain 4: Environment (0–100)	Photovoice participants’ responses	Convergence of photovoice and WHOQOL-BREF	Divergence of photovoice and WHOQOL-BREF
P1	44	50	44	31	‘This is the toilet currently been used by patients who come to this clinic. One can see that it is not accessible to persons using wheelchairs, crutches and those with visual impairment. There is no working tap for water inside to wash your hands after using the toilet. As CBR volunteers we have tried to sensitise the clinic staff to improve and were told that a new toilet is coming but its long now since we complained about it’. (P1)	Physical Health & Environment	-
P2	69	56	19	31	‘I am thankful that CBR volunteers usually visit me to assist me with some household duties. However, since I lost my sight, I have not received proper training on using a white cane, reading Braille, counting money, and basic home duties. I use a stick to move around or my daughter to assist me to go to the shop or move around in the community. I hear that the visually impaired centre in Windhoek provides training to reintegrate persons with visually impairment after they lose their sight. I am requesting the CBR Programme to send me for that training as well. Can’t the CBR Programme arrange a training for money and home orientation for visually impaired so that they are independent in using their financial resources?’ (P2)	Environment & Social	Physical Health
P3	44	56	69	44	‘I am grateful that the CBR volunteers helped me to get this wheelchair a long time ago. However, since the wheelchair was not really of my size, I am having problems using it. It is creating back problems for me. I need a new wheelchair for my size and one which can be used in this sandy area’. (P3)	Physical Health & Environment	-
P4	56	38	44	31	‘This is my child who is deaf. We have challenges in communicating to each other in sign language. Even when he goes out alone he finds it tough to communicate and getting information. Though we have developed our own way of communicating at home, we wish if the family can be provided with sign language training. Even those at places like the clinic, police and school should also receive basic sign language training’. (P4)	Psychological, Social relationships, Environment	-
P5	63	88	50	56	‘This is a project of a person with visual impairment who is doing well with this chicken farming project. This really motivates and shows the community that we can also contribute to the society. However, we have challenges with capital to set up such projects and wish if the CBR Programme can look for benefactors who can help fund us with start-up capital’. (P5)	Psychological	-
P6	50	69	31	50	‘This is the Councillor’s office which is very accessible both for entry and the toilets. We wish if all the public buildings are accessible like this. When I come here I don’t need anyone to assist me and this gives me independence’. (P6)	-	Environment

WHOQOL-BREF, World Health Organization Quality of Life instrument.

### Convergence and divergence of photovoice and World Health Organization Quality of Life responses

Table 4 depicts the relevant information drawn from integrating the photovoice responses and the WHOQOL-BREF instrument scores. For example convergence is shown with P1 (Participant 1) who had low scores on the Environmental and Physical Health domain and also expressed challenges with lack of water to wash hands at local clinic as well as poor accessibility to toilets. Further divergence is shown with P2 (Participant 2) who had a high score on Physical Health, yet the participant expressed dissatisfaction that she has not yet received proper training on using a white cane, reading Braille, counting money and basic home duties since she became visually impaired.

### Feasibility outcomes and critical issues to consider in main study

The overall outcomes were drawn with reference to the feasibility objectives and criteria of the pilot study. The lessons learnt and proposed modifications for the main study are outlined in Table 5.

**TABLE 5:** Feasibility outcomes and proposed modifications.

Main reason	Issue assessed	Lessons learnt	Proposed modifications
1. Process: Assessing the feasibility of the processes that are key to photovoice and WHOQOL-BREF instrument	Participant characteristics	Including caregivers, family members and siblings of persons with disabilities gives a potentially incorrect perspective of experiences and quality of persons with disabilities	Include in criteria: physical disabilities, able to use a camera and describe a picture Exclude in criteria: caregivers, family or siblings of persons with disabilities, person below 18 years of age, intellectual disabilities, mental illness, highly dependent on medical care, HIV positive and previous traumatic war experiences or stressful life circumstances
	Selection of research assistant	Persons with hearing impairment were excluded in the study as both researcher and research assistant had no sign language skills. Eliciting experiences can be affected if researchers are not sensitive to the local culture.	Research assistant to have at least 3 years of CBR experience Research assistant to have at least 2 years working in CBR Programme of that region Should be able to speak a local language of that area Be well versed with local culture Have basic sign language skills
	Retention of participants	Retention rate was 6 out of 8 (75%). Reasons for drop - out could include lack of incentives and poor communication with research assistant	Avail airtime for group leader of participants for constant communication with researcher and research assistant Consider incentives like airtime for mobile phones, certificates of completion and t-shirts/hats
	Understanding by the research assistant on the data collection tools-: photovoice technique and WHOQOL-BREF	Though explained by researcher, the research assistant’s understanding of photovoice technique and WHOQOL-BREF was poor	Research assistants need thorough training and orientation to the photovoice process and WHOQOL-BREF before selecting participants Appendix 3 should be translated into Afrikaans and Oshiwambo
	Adherence to photovoice ethical issues	Participants had challenges getting signatures for providing consent for taking photographs of human beings, as most of the participants and subjects could not read and write	Subjects to be photographed or their caregivers can provide an ‘X’ as indication of signature and then the researcher or research assistant will follow up these subjects to confirm consent Need to request consent for publication from both participants and subjects photographed
2. Resources: Forecast time and resource problems that can occur during the main study	Process time for photography assignment to individual interviewing	Some participants took time to recall why they took the photograph because of the time lag in processing the cameras and interviewing	Cameras should be processed immediately after photography assignment and interviewing should commence soon afterwards
	Establish time needed to fill out the WHOQOL-BREF instrument	It took roughly 40 min to an hour in filling out the instrument as participants were provided with assistance if needed Participants were tired of filling in the instrument following photovoice interviewing	It would be more productive to take a break in between photovoice interviewing and filling in of WHOQOL-BREF instrument
	Type and quantity of language version of the WHOQOL-BREF instruments needed	Most participants could understand the Afrikaans WHOQOL-BREF version	More copies of the Afrikaans or relevant language versions should be printed
	Sign language interpreters	Persons with hearing impairment were excluded because of lack of sign language interpreter	Hire a sign language interpreter for participants with hearing impairment
	Availability and cost of Braille or large print instruments	Braille material was not available for one participant with visual impairment and thus relied on interpretation.	Establish resources and cost for Braille and printing material for the blind and visually impaired participants
	Type of camera to use	Disposable cameras allowed participants a limited number of photographs; they are strong and economical to purchase. Place for processing the cameras was near. The quality of some pictures taken was fair.	Disposable cameras are ideal in rural settings and for photovoice process. Establish the proximity of facilities to processing the disposable cameras. Participants should be trained on photographic techniques to improve picture quality.
	Contingency plans for cameras	One of the participants reported a broken camera. Research assistant had to replace the camera within a short period	Establishing contingency plans in case participants’ cameras are broken or lost before processing
	Distance, transport and time to reach study site	The researcher underestimated the time needed to reach the research site and the type of transport needed	In selecting study site, the researcher should consider type of transport, distance and time needed to reach the site
3. Management: Establishing potential human and data management problems	Challenges of participants during photovoice process and filling in WHOQOL-BREF	Some participants reported lack of transport to reach places of photography Most participants needed privacy to answer some questions on the WHOQOL-BREF instrument especially question 21 asking sexual feelings	Participants should continuously be provided with support during the photovoice process Transport arrangements should be made for some participants Ensure privacy and confidentiality in administering the WHOQOL-BREF instrument.
	Number of photograph assignments and individual interviewing	Two photography assignments and individual interviewing sessions were conducted and this ensured refinement of data	Two or more photograph assignments and individual interviewing sessions are ideal to ensure that data are refined
	Data storage	Researcher stored the data in an encrypted file on the computer	Data will be stored in a secure locked safe and an encrypted file on the computer at the University with the supervisor. Data will be disposed of through shredding after 5 years.
	Data dissemination	Participants’ and subjects’ names were not used and faces were not shown	Participants’ and subjects’ name will not be used and participants’ faces on photographs will not be shown. Participants and subjects were requested for consent to publish the photographs in community gallery, posters, conferences, journals and books.
4. Scientific: Assessment of trustworthiness, response to photovoice and WHOQOL-BREF instrument	Sample size	Six participants were ideal for photovoice process and administering WHOQOL-BREF	Sample of 6–10 is ideal (Wang & Burris 1997). However, to test internal consistency of the WHOQOL-BREF instrument, a sample > 200 is ideal.
	Trustworthiness	Lincoln and Guba model of trustworthiness was applied for the photovoice process. This ensured credibility, transferability, dependability and confirmability	Lincoln and Guba’s model of trustworthiness should be applied with the photovoice process
	Duration of study	This study lasted 2 weeks for photovoice WHOQOL-BREF instrument was administered at end of photovoice process to establish baseline on quality of life	Duration of photovoice process is determined by data saturation. More than one photovoice assignment can allow for saturation. To measure change in quality of life as a result of CBR Programme implementation, the WHOQOL-BREF can be administered over a period of 1 year.
	Multiple study centres	Only one site was utilised	Both photovoice and WHOQOL-BREF have been proposed to be implemented with multiple study sites and compare results
	Feasibility of combining the photovoice process and WHOQOL-BREF instrument	Administering WHOQOL-BREF instrument at end of photovoice process allows for comparison of results and confirmation of photovoice findings with WHOQOL-BREF	Administering WHOQOL-BREF instrument at end of photovoice process allows for comparison of results and confirmation of photovoice findings with WHOQOL-BREF

*Source*: Adapted from Van Teijlingen, E.R, Rennie, A.M, Hundley, V. & Graham, W., 2001, ‘The importance of conducting and reporting pilot studies: The example of the Scottish births survey’, *Journal of Advanced Nursing* 34, 289–295. https://doi.org/10.1046/j.1365-2648.2001.01757.x

WHOQOL-BREF, World Health Organization Quality of Life instrument.

## Discussion

### Feasibility of the processes that are key to implementing photovoice and World Health Organization Quality of Life instrument

Although the scoping review (Shumba & Moodley 2017b) demonstrated that caregivers and siblings of persons with disabilities can be included in determining experiences, this study demonstrated that including caregivers and persons with disabilities can affect the outcomes, especially with respect to experiences elicited and measurement of their respective qualities of life using the WHOQOL-BREF instrument. Caregivers and persons with disabilities have different experiences posing challenges in equating their assessments related to quality of life. Furthermore, caregivers introduced issues that are more important to them than persons with disabilities to whom they provide assistance.

The selection of research assistant is key in ensuring quality purposive sampling, competence with local language and retention of participants. In this pilot study, the research assistant was a Senior Community Liaison Officer who had only 1 year experience working with the Groot Aub CBR Programme. This created challenges with purposive sampling as the research team depended heavily on the local persons with disabilities to identify participants. Thus, some of the key attributes in selecting the research assistant in main study are: at least 3 years of CBR experience, at least 2 years working in CBR Programme of that region, should be able to speak a local language of that area and be well versed with local culture and basic sign language skills.

Furthermore, the pilot study excluded persons with hearing impairment as none from the research team had basic sign language skills. Persons with hearing impairment may be good with the photography component, but it may be difficult to engage them in a critical discussion (Jurkowski 2008) as they require sign language interpretation. Another group that this pilot study excluded included persons with intellectual and severe disabilities as a result of the extensive assessments needed and the uncertainty of ensuring that their interest and reasonable accommodation are genuinely taken into account (Ware 2004).

Participants had challenges getting signatures and consent for taking photographs of human beings as most of the participants and subjects could not read and write as indicated in other studies (Akkerman et al. 2014). Consent forms in most cases are written in higher language required by review boards, which may be difficult for participants to understand (Lennox et al. 2005). The researcher translated and explained the consent forms for subjects to be photographed. Furthermore, the researcher recommended the participants to acquire consent by using an ‘X’ from the subjects to be photographed or their caregivers as an indication of signature and then the researcher or research assistant then made follow-ups of these subjects to confirm they gave consent.

The ‘SHOWeD’ technique suggested by Wang in 1999 was not successful with the pilot study as most participants indicated that it was rigid and provided only limited interpretation and exploration of their experiences. In similar studies, McIntyre (2003) also reported that the ‘SHOWeD’ technique had challenges in limiting the participants’ interpretation of their photographs. McIntyre suggested the use of photographer’s instinct in selecting photographs. These photographs are then interpreted and analysed based on personal questions. The researcher then adopted the approach by McIntyre and utilised personal questions. Furthermore, participants were not requested to document their experiences on paper as most participants were not articulate enough to explain clearly their experiences let alone write them concisely.

The main modification of the original photovoice process that was piloted was replacement of collective group discussion with one-on-one interviews in the photovoice analysis. This study revealed that one-on-one interviews with persons with disabilities provided confidentiality allowing participants to express themselves freely without influence from others and preventing the perspectives of participants from being shaped by others (Jurkowski 2008). Furthermore, it provides a more personal setting that facilitates sharing of deeper thoughts with the result being richer interview data (Newman 2010). Also, individual interviewing allowed the researcher to visit each participant’s home and this alleviated the issue of transportation, as most of the participants had mobility or transportation challenges.

### Time projection and anticipated resource problems

To ensure success of the main study, it was key to note the process time for the photography assignment, individual interviewing and to establish the time needed to fill out the WHOQOL-BREF instrument. Although duration of the photovoice method varies according to the objectives of the project, it is important to note that photovoice is more time consuming than other traditional research as it requires developing relationships and trust with participants (Jurkowski 2008). To this end, the researcher should invest a reasonable amount of time and be patient with participants. This study establishes that cameras should be processed immediately after the photography assignment and interviewing should commence as soon as possible so that participants can recall why they took the photo. The WHOQOL-BREF instrument indicated a considerable amount of time required to complete it. Thus, it is ideal to take a short break in between photovoice interviewing and filling in of WHOQOL-BREF instrument to allow participants to refresh.

Photovoice can be used with any disability type provided reasonable accommodation is provided (Shumba & Moodley 2017b). The anticipated resource problems to cater for reasonable accommodation of selected participants include sourcing sign language interpreters and identifying the sourcing and cost of Braille or large print of WHOQOL-BREF instruments. Additional costs to be noted include type of camera to use (digital or disposable-film camera), contingency cameras in case participant camera is lost or broken and distance and transport cost to reach study site. The researcher learnt that it is advisable to actively engage participants throughout the project to increase retention and to avail airtime for mobile phone use to the group leader of participants for constant communication with researcher and research assistant. Furthermore, to help ensure commitment, participants could be given incentives including t-shirts or hats during their photography assignment and certificates of completion at the end of project.

### Potential human and data management problems

Final stories, themes and theories in photovoice are determined by saturation of data. However, reaching data saturation in the photovoice process can be attained in different ways. It is suggested that participants should be given an opportunity to comment on all photographs that they had taken as limiting the participants to selecting best photographs can hinder the collection and analysis of a wide range of issues (Newman 2010). On the other hand, participants can be given a specified or maximum number of photographs to take in a given period. This allows participants to be critical or selective on which photographs to take. For this pilot study, participants were given disposable cameras (27 exposures film) thus limiting the number of photographs instead of digital cameras, which allow for taking more photographs. Additionally, participants should be trained on photographic techniques to improve picture quality.

Participants were allowed one round of photography assignment and requested to comment on their photographs that best describe their experiences. This was an effective approach as it allowed participants to be critical on issues they photographed and saved time on interviewing and resources needed to process the photographs. However, the researcher noted that participants could be given more than one round of photography assignment. Giving participants more than one round of photography assignment can afford them the opportunity to reflect on their issues and refine their findings. Furthermore, it can allow for saturation when both the researcher and participant can agree that there are no new issues emerging.

The study included the category ‘photographs not taken’ (i.e. photographs participants thought of taking, but failed to, because of other reasons) (Lassetter, Mandleco & Roper 2007) in the feedback session and this produced rich data. One main reason that some participants failed to take some photographs was lack of transport to take photographs of public places where they receive services. Thus, one of the recommendations for the main study is to provide transport arrangements to sites of photography as per participants’ request.

The original conceptualisation of photovoice process by Wang and Burris (1997) allows for group selection of final themes before organising a community gallery. However, Shumba and Moodley (2017b) argued that participants should be given the flexibility to accept or decline proceeding further with the photovoice process after taking photographs. This pilot study gave participants an opportunity to accept or decline the offer to participate in a final group discussion and photo gallery. One participant declined citing confidentiality issues and fear of being victimised by the authorities and family members. Persons with disabilities often lack self-esteem and confidence in representing themselves in a public platform.

Photovoice potentially generates a lot of data, and thus it is important to decide on storage of data and disposal. Several issues need to be taken into account, including: awareness of data protection legislation, secure storage (encryption, lockable locker), awareness of security standards for online or cloud data collection and storage, publication of data and disposal of data (European Commission 2009). When disseminating research findings, it is critical to observe ethical principles including consent for publication of findings. In this pilot study, names of participants and subjects were not used and their faces on photographs were not shown. Furthermore, participants and subjects were asked for consent to publish the photographs in a community gallery, posters, conferences, journals and books.

### Trustworthiness and response to photovoice and World Health Organization Quality of Life assessment

To determine trustworthiness and data saturation, two or more photography assignments and individual interviews are ideal. However, despite photovoice enabling participants to mitigate communication problems, some of the participants had limited vocabulary and limited articulations skills to explore their experiences and this raised queries on the correctness or accuracy of interpretations of some photographs. In some instances, there was a discrepancy between what the photograph appears to identify and what the participant explained in the interview.

Another issue that is critical to the photovoice process is sample size. The sample of 6–10 was deemed ideal (Wang & Burris 1997). A scoping study (Shumba & Moodley 2017b) reviewed sample sizes from as small as 1 participant to 82 participants. This indicates that photovoice can be used with a range of sample sizes. However, Wang (1999) suggested a group of 6–10 participants as an ideal size for the photovoice method. Such a sample size is considered large enough to offer in-depth experiences and ideas, yet also allows enough time for each participant to contribute in a meaningful way. In addition, groups of this size are small enough so that members are able to feel safe in sharing experiences and taking part in discussions (Palibroda et al. 2009). The current study used a sample size of eight, but only six participants completed the study.

The photovoice process of this study lasted 2 weeks. Duration of photovoice process is determined by data saturation and more than one photovoice assignment can allow for saturation (Shumba & Moodley 2017b). The main study can focus on not only establishing baseline quality of study but also consider longitudinally measuring change in quality of life. The WHOQOL-BREF Group (WHOQOL-BREF Group 1996) proposed some uses of WHOQOL-BREF instrument including establishing baseline scores in a range of areas, determining changes in quality of life over the course of interventions, research and policymaking. Other uses that can be proposed for the main study are determining changes in quality of life at impact evaluation intervals and providing evidence for policy changes (WHOQOL-BREF Group 1996).

Although the WHOQOL-BREF instrument offers a generic measurement on quality of life, WHO developed an additional module to the WHOQOL-BREF called ‘WHOQOL-Dis’ to measure specific aspects on quality of life of persons with physical and intellectual disabilities. Bredemeier et al. (2014) stated that the WHOQOL-Dis is a measurement option for quality of life and thus recommended joint administration with the WHOQOL-BREF instrument. To this end, the main study can potentially jointly administer the WHOQOL-BREF and WHOQOL-Dis instruments to gain both a generic and an in-depth understanding of the quality of life of persons with disabilities.

### Overall evidence of feasibility

This study demonstrated that photovoice is a flexible method that can be used with a variety of disabilities and has the potential of being combined with the WHOQOL-BREF instrument. Furthermore, there is need to provide continuous support during the photovoice process and in completing the WHOQOL-BREF instrument. Photovoice allowed most participants who were not confident to verbally communicate to meaningfully participate in the study. Administering the WHOQOL-BREF instrument at the end of the photovoice process can measure both the quality of life of persons with disabilities and indicate the convergence and divergence in the two data collection methods as shown in Table 4. The study demonstrated a stronger convergence than divergence of the two methods (Table 4). Matching the photovoice responses to the scoring on WHOQOL-BREF instrument consistently identified issues that were important to persons with disabilities. Furthermore, integrating the two methods provided an adequate representation of the concerns and issues of persons with disabilities regarding the CBR Programme. However, divergence of outcomes of photovoice and WHOQOL-BREF instrument indicates that caution should be taken when combining both methods because of the diverse nature of persons with disabilities. The participants were requested to identify issues related to all the five components of the WHO CBR Matrix (health, education, livelihood, social relationship and empowerment). There is a potential to explore in-depth issues when the methods are combined to focus on a specific component, for example, health. When there is a specific activity to focus on, the combination is likely to produce in-depth rich data.

## Conclusion

The findings from this study offer insights and considerations that can potentially be utilised in future large scale studies. The study identified critical issues related to the process of using the photovoice process with persons with disabilities and administering WHOQOL-BREF instrument on a large scale including ethical considerations, human and financial resource issues, time forecast, trustworthiness and data management. Furthermore, the study demonstrated the potential of combining photovoice method and WHOQOL-BREF questionnaire. However, the combination of these methods should be made cautiously given the diverse nature of disability.

Noteworthy is that the ‘formal’ WHOQOL-BREF instrument identified complex issues related to quality of life and the ‘informal’ photovoice method brings to light concrete issues related to everyday life that are usually left out by researchers and discussed less with family members and caregivers. A range of good outcomes that arose from integration of photovoice responses and WHOQOL-BREF instrument score consistently depicted issues that were important to participants. The feasibility of utilising each method alone and in combination offered valuable insights on future conceptual framing of CBR Programme evaluation. This conceptual framing will allow CBR practitioners to appreciate how these two methods contribute to a rigorous process of CBR Programme evaluation. The lessons learnt in this pilot study will increase awareness on the potential pitfalls and optimise the use of photovoice and the WHOQOL-BREF instrument. A larger cohort study may consider implementing photovoice and both WHOQOL-BREF instrument and WHOQOL-Dis on multiple study sites and be able to compare results, considering geographical or demographic variables. Future research may also consider further investigation and evaluation of the use of photovoice as a disability research tool and WHOQOL-BREF instrument with reference to the WHO CBR Matrix (WHO et al. 2010) and its potential use in CBR monitoring and evaluation. Gee et al. (2000) argued that quality of life assessments are critical in decision-making and resource allocations for programme interventions.

## Appendix 1: Information sheet and consent to participate in study.

Date:

Good day

My name is Tonderai Washington Shumba with the details listed below:

**Table:**

**Academic Affiliation:**	University of KwaZulu-Natal College of Health Sciences South Africa
**Degree Programme:**	Doctor of Philosophy in Public Health Medicine (Student)
**Mobile:**	0813631898
**Email:**	shumbatw@gmail.com

You are being invited to consider participating in a PhD study that involves developing a monitoring and evaluation tool that can be used to elicit the experiences of people with disabilities on the Community Based Rehabilitation Programme (CBR) in Namibia and to improve their quality of care. The main aim of this study is to improve the quality of life of people with disabilities by monitoring the care and quality of services provided. The study will involve 4 phases of which you are being invited for the pilot phase (third phase) of the study.

The study is expected to enroll a total of 36 participants over different phases. For this third phase there will be a total of 8 participants from Khomas region. Phase 1 and 4 will have 12 and 16 participants respectively. Phase 1 and 4 participants will be drawn from the following regions: Khomas, Hardap and Otjozondjupa.

The purpose of this information sheet and consent form is to provide the information needed to help you decide whether or not to participate in this research project. Included is information on the importance and benefits of the research, possible risks, and your rights as a volunteer participant. When all your questions have been answered, you can decide if you want to be in the study or not. This process is called ‘informed consent’. Please read the form carefully and feel free to ask any questions you may have.

### Purpose of the Pilot research project

The purpose of the pilot phase in which you are invited is to pilot a photovoice tool for monitoring and evaluating experiences of people with disabilities on the CBR programme in Namibia and assess improvements in quality of life using a World Health Organization Quality of Life Questionnaire. The photovoice process will be used to gather information. The purpose of taking photos, and asking for an explanation of the photo, is to help you tell your story with your own pictures. There are three goals of this research –

- To enable you as a person with a physical disability to record and reflect on disability issues which have /have not been addressed by your Community Based Rehabilitation programme.
- To promote discussion about important disability issues through taking photographs and talking about them.
- To engage policymakers about disability issues that are important to you as a person with physical disability.

It is hoped that the results of this pilot research will help us adapt tools that can assist us to better understand issues surrounding Community Based Rehabilitation as a programme. This information may be of assistance in improving the quality of life for persons with disabilities.

### Procedures

If you choose to be in this study, both data collection and analysis will last for 1 month as described below:

You will be asked to attend a one day training workshop about photovoice method, use of cameras, and the responsibility of taking photographs. In the workshop we will discuss ethics, ways of seeing photographs, and the idea of giving back photographs to the community members as a way of expressing appreciation and respect. During the workshop you will be asked questions like ‘What is an acceptable way to approach someone to take their photograph?’, ‘Should pictures be taken of people without their knowledge?’, ‘What kind of responsibility does carrying a camera involve?’, ‘What types of photographs do you think are private or personal?’ and ‘To whom might you wish to give photographs, and what might be the implications?’.

You will be provided with a camera and film for the research. The researcher will develop your pictures and provide you with copies for discussions. The best photographs will be selected for the research project.

The photovoice process will ask that you spend 1 month taking photos with a digital camera and then deliver the cameras back to us every week. We will develop your photos and return them to you. We will then ask you to select and reflect in writing on six of your pictures that you believe are most meaningful in their description of the work of Community Based Rehabilitation and that you would want to share with a broader audience. You will return the photos and reflections to us. You will then participate in a one day learning workshop at the end of each week. This will engage you and other participants in the photovoice process through a facilitated discussion and analysis. During this 1-day session, we may be audio taping and video recording the conversations and taking field notes. At any time, you can request that the recorders be turned off. The recordings and transcripts will be kept in a locked file cabinet, and your identity (if you choose not to be identified by name) will not be disclosed (we will use ‘site participant’).

### Potential risks, stresses, or discomfort

Some people may feel taking pictures is an invasion of their privacy. Some people feel a little self-conscious when they are photographed. Some people may not want to have their photo taken. No photographs identifying specific individuals will be released without a separate written consent of the photographer and the identified individuals. No picture is worth taking if it makes someone feel uncomfortable.

Because of the small number of participants (8), identity might be discerned; therefore, only limited confidentiality can be guaranteed. However, your privacy will be protected to the maximum extent allowable by law. Please know that participation in this project is voluntary and that you may choose at any time not to participate.

### Benefits

This research project aims to benefit the CBR programme and the community as a whole. You may not directly benefit from taking part in this study; however, we believe that this research will give you a creative way to express yourself by identifying disability issues that are important to you.

### Other information

Information about you is confidential. The recordings and transcripts will be kept in a locked file cabinet, and your identity (if you choose not to be identified by name) will not be disclosed (we will use ‘site participant’). Study information will be coded and linked between your name and the code in a separate, secured place until the final document of the research is ready for submission. If the results of this study are published or presented, your name will not be used. Although the researcher will take every precaution to safeguard your confidentiality and privacy, it cannot guarantee.

This study has been ethically reviewed and approved by the UKZN Humanities and Social Sciences Research Ethics Committee (approval number HSS/0646/015D) and Ministry of Health and Social Services in Namibia (approval number17/3/3).

In the event of any problems or concerns/questions you may contact the researcher at (provide contact details) or the UKZN Humanities & Social Sciences Research Ethics Committee, contact details as follows:

HUMANITIES & SOCIAL SCIENCES RESEARCH ETHICS ADMINISTRATION

Research Office, Westville Campus

Govan Mbeki Building

Private Bag X 54001

Durban 4000

KwaZulu-Natal, SOUTH AFRICA

Tel: 27 31 2604557- Fax: 27 31 2604609

Email: HSSREC@ukzn.ac.za

Taking part in this study is voluntary. You can stop at any time. It is anticipated that participants will avail their time only and not incur any costs as a result of participation in the study. If there are transport costs incurred, the researcher will reimburse. There will be a flat N$700 to be given at the end of the research as a token of appreciation. In the event of refusal/withdrawal of participation the participants will receive a pro-rata benefit calculated out of the N$700. In addition the researcher will give you a t-shirt and umbrella for participating in this research. You are however requested to inform the researcher of your intention to withdraw to ensure orderly exit from the group. The researcher reserves the right to terminate participation in cases of lack of commitment.

### CONSENT TO PARTICIPATE IN PILOT PHOTOVOICE STUDY

I (……………………Name) have been informed about the PhD study entitled ‘Developing a monitoring and evaluation tool that can be used to elicit the experiences of people with disabilities on the Community Based Rehabilitation Programme (CBR) in Namibia and to improve their quality of care’ by Mr Tonderai Washington Shumba.

I understand the purpose and procedures of the study.

I have been given an opportunity to answer questions about the study and have had answers to my satisfaction.

I declare that my participation in this study is entirely voluntary and that I may withdraw at any time without affecting any of the benefits that I usually am entitled to.

I have been informed about any available incentives and how they will be issued to me.

If I have any further questions/concerns or queries related to the study I understand that I may contact the researcher at: mobile number: 0813631898 or email: shumbatw@gmail.com

If I have any questions or concerns about my rights as a study participant, or if I am concerned about an aspect of the study or the researchers then I may contact:

HUMANITIES & SOCIAL SCIENCES RESEARCH ETHICS ADMINISTRATION

Research Office, Westville Campus

Govan Mbeki Building

Private Bag X 54001

Durban 4000

KwaZulu-Natal, SOUTH AFRICA

Tel: 27 31 2604557 - Fax: 27 31 2604609

Email: HSSREC@ukzn.ac.za

#### Additional consent:

I hereby provide consent to:

1. Audio-record my interview / focus group discussion    YES / NO
2. Video-record my interview / focus group discussion    YES / NO
3. Use of my photographs for research purposes      YES / NO

**Table:**

____________________ Signature of Participant	____________________ Date
____________________ Signature of Witness (Where applicable)	_____________________ Date
____________________ Signature of Translator (Where applicable)	_____________________ Date

## Appendix 2: Photo Release Form (Subject informed consent).

### Informed consent form (For person to be photographed)

(to be translated into local languages)

### Photograph consent form

I, __________________________________________, hereby permit photographs of myself to be used in research projects about experiences of people with physical disabilities. This research is being carried out in conjunction with the Community-Based Rehabilitation Programme. I give permission to the investigator and photographers to use these photographs for use in all relevant Community Based Rehabilitation research for the purposes of education, communication, promotion and increasing understanding of disability issues.

I have read and understand this consent form and agree to its terms knowingly and voluntarily.

## Appendix 3: Foto Vrylating Vorm.

### Foto toestemingsvorm

Ek, ………………………………., gee hiermee toestemming dat ‘n foto van my geneem mag word vir die gebruik van die navorsing oor die ervaring van mense met liggaamlike gestremhede. Hierdie navorsing word gedoen in samewerking met die gemeenskapsgebaseerde rehabiliterings program. Ek gee toestemming vir die navorser en fotograwe om die foto’s te gebruik in alle toepaslike gemeenskapsgebaseerde rehabiliterings navoring vir opvoedkundige, kommunikasie en bevorderings doeleindes, as ook om begrip en kennis oor gestremdhede te verhoog.

Ek het hierdie toestemmingsvorm gelees, verstaan die inhoud daarvan en aanvaar die bepalings ten voll en vrywilliglik.

## Appendix 4: Consent for publication.

### CONSENT FORM

I …………………………….………………………………..…………….……………………..…………… [Name] give my consent for information about myself/my child or ward/my relative (circle as appropriate) to be published in African Journal on Disability (Manuscript 418: Corresponding author: Tonderai Washington Shumba].

I understand that the information will be published without my/my child or ward’s/my relative’s (circle as appropriate) name attached, but that full anonymity cannot be guaranteed.

I understand that the text and any pictures or videos published in the article will be freely available on the internet and may be seen by the general public. The pictures, videos and text may also appear on other websites or in print, may be translated into other languages.

Signing this consent form does not remove my rights to privacy.

Name…………………………….

Date…………………………….

Signed…………………………….

Author name…………………………….

Date…………………………….

Signed…………………………….

## Appendix 5: Photovoice ethics: safety, impact, and obligation.

Photovoice is, by design, intended to include participants in participatory inquiry. They become documentary photographers at their site; their objective is to take pictures of activities, events, symbols, and people (photo subjects) that best respond to the framing (trigger) questions. The impact of this work can extend to include:

- The photovoice photographers,
- The photo subjects, and
- The broader community

### Community gallery

Although safety and ethical considerations will vary across situations and rarely lend themselves to standard solutions, we can benefit from consideration of the following issues and questions.

### Safety

Photovoice participants are asked to photograph the work of their community. They may document elements of strength and issues of concern. Recording these elements for public dissemination could have negative repercussions for the participant – as the photo is being taken or after the photo and explanation of it have been disseminated. Here are some concerns and what we will instruct photovoice participants to do in practice.

### Key concerns

- Potential risks to photovoice photographers from putting themselves in dangerous settings or situations.
- Potential risks to photovoice photographers from photo subjects.
- Potential risks to photovoice photographers from being identified in connection with their photos and stories.

### Your practice

Give careful thought to the context and content of your photos – the communities in which you live, the issues you will be exploring and the situations you might get into while documenting your work.

- Because you know your neighbourhoods better than we do, we encourage you to use your street sense.
- ‘Shooting smart’ – maintaining your personal safety – is of highest priority. No photo is worth personal danger.
- Remember that there are alternative ways to present issues (e.g. through abstract representation).
- Take your photos in public spaces (from which participants can photograph without being seen as trespassing) versus private property.

### Subjects of photographs

The evaluation team and the photovoice participants have an ethical responsibility to their photo subjects. We want to emphasise that photovoice photographs are meant for dissemination. For this reason, there is no point in taking photos that cannot be shown for lack of the subject’s permission through the release form. Here is our key concern and what we instruct photovoice participants to do in practice.

### Key concerns

Potential risks to photo subjects from being identified in connection with particular situations or activities in photos.

### Your practice

As a documentary photographer, you must respect the privacy of others. If someone does not want his or her picture taken, don’t take it.

- It is essential that photo subjects sign a release form to be photographed. We have included forms. For children or youth under the age of 18, you will need approval from a parent or guardian. This is provided for on the release form. Please make more copies if you need them.
- Please emphasise to photo subjects that the photographs are meant for dissemination. Photos cannot be shown without a subject’s release.
- Again, there are ways to portray issues of concern that don’t require showing individuals.

### Impact on your community Key concerns

- Potential risks to your community as a whole through generating conflict around issues or negative image.

### Your practice

Because of your background using a number of dissemination tools, we are confident that you understand the importance of weighing potential for collective good against potential for both individual and collective harm.

### Obligation of the evaluation team Key concerns

- The photovoice process puts the evaluation team in a close partnership with site participants. The effectiveness of our work is based on bonds of trust and our commitment that participant stories and voices be meaningful.
- At the same time, we know that you have invested in the photovoice process and data. Because of the many potential uses for these data, we will share stories in a variety of ways for a variety of purposes.

### Your practice

Because of your background using a number of dissemination tools, we are confident that you understand the importance of weighing potential for collective good against potential for both individual and collective harm.

- We will strive to build a participatory component through ongoing phases of analysis.
- You will be a part of the decision making process in how the photos and stories will be disseminated.
- We will strive to balance agendas through finding ‘both/and’ solutions and multiple avenues for dissemination that meet the needs of various stakeholders.

## Appendix 6: WHOQOL-BREF instrument.

### ABOUT YOU

I.D. number:

Before you begin we would like to ask you to answer a few general questions about yourself: by circling the correct answer or by filling in the space provided.

**Table:**

What is your **gender**?	Male		Female		
What is your **date of birth**?	__________	/	__________	/	
	Day	/	Month	/	Year
What is the highest **education** you received?	None at all		Primary school		Secondary school Tertiary
What is your **marital status**?	Single	/	Separated	/	Married /
	Divorced	/	Living	as	married /
	Widowed				
Are you currently **ill**?	Yes No				

If something is wrong with your health what do you think it is?

### Instructions

This assessment asks how you feel about your quality of life, health, or other areas of your life.

**Please answer all the questions.** If you are unsure about which response to give to a question, **please choose the one** that appears most appropriate. This can often be your first response.

Please keep in mind your standards, hopes, pleasures and concerns. We ask that you think about your life **in the last 2 weeks.** For example, thinking about the last 2 weeks, a question might ask:

**Table:**

	Do you get the kind of support from others that you need?	Not at all 1	Not much 2	Moderately 3	A great deal 4	Completely 5

You should circle the number that best fits how much support you got from others over the last 2 weeks. So you would circle the number 4 if you got a great deal of support from others as follows.

**Table:**

	Do you get the kind of support from others that you need?	Not at all 1	Not much 2	Moderately 3	A great deal 4	Completely 5

You would circle number 1 if you did not get any of the support that you needed from others in the last 2 weeks. Please read each question, assess your feelings, and circle the number on the scale for each question that gives the best answer for you.

### THE WHOQOL-BREF

**Table:**

		Very poor	Poor	Neither poor nor good	Good	Very good
1 (G1)	How would you rate your quality of life?	1	2	3	4	5

**Table:**

		Very dissatisfied	Dissatisfied	Neither satisfied nor dissatisfied	Satisfied	Very satisfied
2 (G4)	How satisfied are you with your health?	1	2	3	4	5

The following questions ask about **how much** you have experienced certain things in the last 2 weeks.

**Table:**

		Not at all	A little	A moderate amount	Very much	An extreme amount
3 (F1.4)	To what extent do you feel that (physical) pain prevents you from doing what you need to do?	1	2	3	4	5
4 (F11.3)	How much do you need any medical treatment to function in your daily life?	1	2	3	4	5
5 (F4.1)	How much do you enjoy life?	1	2	3	4	5
6 (F24.2)	To what extent do you feel your life to be meaningful?	1	2	3	4	5

**Table:**

		Not at all	A little	A moderate amount	Very much	Extremely
7 (F5.3)	How well are you able to concentrate?	1	2	3	4	5
8 (F16.1)	How safe do you feel in your daily life?	1	2	3	4	5
9 (F22.1)	How healthy is your physical environment?	1	2	3	4	5

The following questions ask about **how completely** you experience or were able to do certain things in the last 2 weeks.

**Table:**

		Not at all	A little	Moderately	Mostly	Completely
10 (F2.1)	Do you have enough energy for everyday life?	1	2	3	4	5
11 (F7.1)	Are you able to accept your bodily appearance?	1	2	3	4	5
12 (F18.1)	Have you enough money to meet your needs?	1	2	3	4	5

**Table:**

		Not at all	A little	Moderately	Mostly	Completely
13 (F20.1)	How available to you is the information that you need in your day-to-day life?	1	2	3	4	5
14 (F21.1)	To what extent do you have the opportunity for leisure activities?	1	2	3	4	5

**Table:**

		Very poor	Poor	Neither poor nor good	Good	Very good
15 (F9.1)	How well are you able to get around?	1	2	3	4	5

The following questions ask you to say how **good or satisfied** you have felt about various aspects of your life over the last 2 weeks.

**Table:**

		Very dissatisfied	Dissatisfied	Neither satisfied nor dissatisfied	Satisfied	Very satisfied
16 (F3.3)	How satisfied are you with your sleep?	1	2	3	4	5
17 (F10.3)	How satisfied are you with your ability to perform your daily living activities?	1	2	3	4	5
18 (F12.4)	How satisfied are you with your capacity for work?	1	2	3	4	5
19 (F6.3)	How satisfied are you with yourself?	1	2	3	4	5
20 (F13.3)	How satisfied are you with your personal relationships?	1	2	3	4	5
21 (F15.3)	How satisfied are you with your sex life?	1	2	3	4	5
22 (F14.4)	How satisfied are you with the support you get from your friends?	1	2	3	4	5
23 (F17.3)	How satisfied are you with the conditions of your living place?	1	2	3	4	5
24 (F19.3)	How satisfied are you with your access to health services?	1	2	3	4	5
25 (F23.3)	How satisfied are you with your transport?	1	2	3	4	5

The following question refers to **how often** you have felt or experienced certain things in the last 2 weeks.

**Table:**

		Never	Seldom	Quite often	Very often	Always
26 (F8.1)	How often do you have negative feelings such as blue mood, despair, anxiety, depression?	1	2	3	4	5

Did someone help you to fill out this form? …………………………………………………………………..

How long did it take to fill this form out? ……………………………………………………………………..

Any other comments?

**THANK YOU FOR YOUR HELP**
